# Postoperative pulmonary complications after high-altitude thoracic surgery requiring one-lung ventilation: a single-centre retrospective cohort study

**DOI:** 10.1186/s12871-026-04139-6

**Published:** 2026-08-08

**Authors:** Yanqing Luo, Ronghui Li, Laba Ciren, Xingyue Su, Haiyang Ding, Qi Yan

**Affiliations:** 1 Department of Anesthesiology, Peking University People’s Hospital, Beijing, China; 2 China-Japan Friendship Clinical Medical College, Peking University Health Science Center, Beijing, China; 3 Tibet Autonomous Region People’s Hospital, Lhasa, Xizang Autonomous Region China

**Keywords:** High altitude, Thoracic surgery, General anaesthesia, One-lung ventilation, Postoperative pulmonary complications, Logistic regression

## Abstract

**Background:**

Postoperative pulmonary complications (PPCs) are common adverse outcomes after thoracic surgery and one-lung ventilation (OLV). Their reported incidence varies with endpoint definition and ascertainment. Lower inspired oxygen partial pressure may alter perioperative oxygenation at high altitude, but direct evidence from thoracic OLV cohorts remains limited. We described PPC occurrence and evaluated associated patient and perioperative factors in a high-altitude thoracic OLV cohort.

**Methods:**

This single-centre retrospective cohort included adults undergoing thoracic surgery with general anaesthesia and OLV at Tibet Autonomous Region People’s Hospital (Lhasa, approximately 3,650 m). The primary outcome was an in-hospital composite outcome comprising postoperative pneumonia, imaging-confirmed atelectasis, acute respiratory distress syndrome (ARDS), postoperative respiratory failure, initial postoperative ventilatory support lasting more than 48 h, or reintubation, with events assessed within the first 7 postoperative days. The primary multivariable logistic regression analysis used multiple imputation by chained equations and included 14 covariates. Complete-case and no-BMI multiple-imputation models were sensitivity analyses. Marginally standardised risk ratios (RRs) and average marginal effects (AMEs) complemented odds ratios (ORs).

**Results:**

Among 492 patients, 211 developed PPCs (42.9%), mainly atelectasis (141, 28.7%) and pneumonia (113, 23.0%). In the primary model, ASA physical status at least III (OR 2.37, 95% confidence interval [CI] 1.32–4.26; *P* = 0.004), preoperative anaemia (OR 2.74, 95% CI 1.56–4.81; *P* < 0.001), surgical duration per 1-hour increase (OR 1.40, 95% CI 1.17–1.68; *P* < 0.001), and bronchial blocker use (OR 2.81, 95% CI 1.33–5.90; *P* = 0.007) remained associated with PPCs after multivariable adjustment. Corresponding RRs for these four factors were 1.45, 1.52, 1.16, and 1.51, and AMEs were + 18.1, + 20.6, + 6.8, and + 21.0% points, respectively. The directions and statistical significance of these associations were consistent in sensitivity analyses.

**Conclusions:**

In this single-centre high-altitude thoracic OLV cohort, PPCs occurred in 42.9% of patients and were predominantly atelectasis and pneumonia. After multivariable adjustment, ASA physical status ≥ III, preoperative anaemia, longer surgical duration, and bronchial blocker use remained associated with PPCs. The bronchial-blocker estimate is susceptible to confounding by indication. Without a low-altitude comparator or granular ventilation data, the findings should be interpreted as observational, hypothesis-generating signals rather than causal effects of altitude or lung-isolation devices. External validation is required.

**Supplementary Information:**

The online version contains supplementary material available at https://doi.org/10.1186/s12871-026-04139-6.

## Background

 Postoperative pulmonary complications are important adverse outcomes after thoracic surgery and affect recovery, length of stay, and perioperative prognosis. They commonly include atelectasis, pneumonia, respiratory failure, reintubation, and prolonged mechanical ventilation. Reported incidence varies widely because studies use different populations, observation windows, and diagnostic criteria: approximately 5% in large mixed surgical cohorts, 2%-19% in commonly reported non-cardiothoracic surgical populations, and higher rates in thoracic surgery and OLV-related populations [1–3]. During thoracic surgery, operative-lung collapse, lateral positioning, general anaesthesia, mechanical ventilation, and surgical manipulation may reduce functional residual capacity, promote alveolar collapse and secretion retention, and alter ventilation-perfusion matching [1, 4, 5].

Most PPC risk studies have been conducted in low-altitude or mixed surgical populations. The ARISCAT study provides an established framework for perioperative risk assessment [2], and recent multicentre thoracic studies have evaluated the association between oxygen exposure during OLV and PPCs [3]. Lower inspired oxygen partial pressure is a defining feature of high altitude [6], but the absence of directly comparable low-altitude cohorts precludes assuming an independent causal altitude effect. We therefore described the occurrence and composition of PPCs and evaluated patient and perioperative factors associated with PPCs in a single-centre high-altitude thoracic OLV cohort.

## Methods

### Study design and participants

**Fig. 1:**
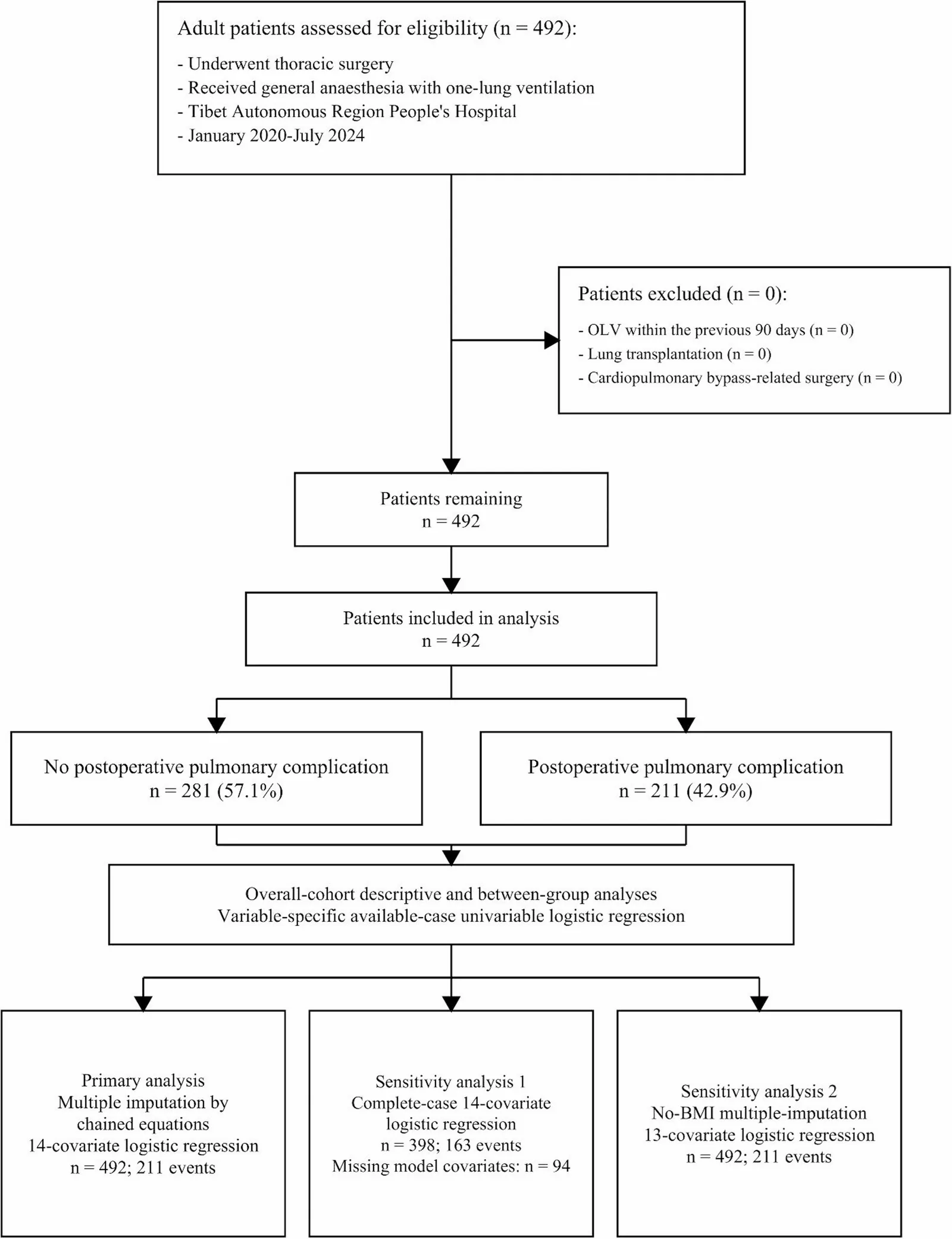
Flow diagram of patient selection and analysis. A total of 492 patients were assessed for eligibility; none were excluded, and all 492 were included in the study cohort. Of these, 281 did not develop PPCs and 211 developed PPCs

This single-centre retrospective cohort study was reported in accordance with the Strengthening the Reporting of Observational Studies in Epidemiology (STROBE) statement [7]. The study was conducted at Tibet Autonomous Region People’s Hospital in Lhasa, China, at approximately 3,650 m above sea level. We assessed 492 adult patients who underwent thoracic surgery with general anaesthesia and OLV between 1 January 2020 and 31 July 2024. The inclusion criteria were age at least 18 years, thoracic surgery, general anaesthesia, and intraoperative OLV. The exclusion criteria were non-thoracic surgery, absence of OLV, OLV within the preceding 90 days, lung transplantation or cardiopulmonary-bypass procedures, inability to verify key information, or an indeterminate primary outcome. All 492 assessed patients met the eligibility criteria; no patients were excluded, and 492 were included in the final cohort (Fig. 1).

This study was approved by the Medical Ethics Committee of Tibet Autonomous Region People’s Hospital (approval no. ME-TBHP-24-KJ-101; 20 July 2025). The study was conducted in accordance with the Declaration of Helsinki. The committee waived the requirement for informed consent because the study used de-identified retrospective data.

### Outcome definition

The primary outcome was an in-hospital composite PPC endpoint, defined as the occurrence within the first 7 postoperative days of any of the following: postoperative pneumonia, imaging-confirmed atelectasis, acute respiratory distress syndrome (ARDS), postoperative respiratory failure, initial postoperative ventilatory support lasting more than 48 h, or reintubation [2, 3]. The radiographic definition of atelectasis was informed by Canet et al. [2], while the overall PPC framework and definitions of the other components, including ARDS, were based principally on Douville et al. [3]. Pleural effusion and pneumothorax were not included. Pre-existing pneumonia was treated as a baseline covariate, whereas newly diagnosed postoperative pneumonia was an outcome component. PPC components were retrospectively ascertained from perioperative records, imaging reports, complication records, treatment records, and structured outcome fields. No independent blinded endpoint adjudication was performed. Detailed operational definitions and observed component frequencies are provided in Supplementary Appendix S1.

### Variables and coding

Collected variables included age, sex, ethnicity, body mass index (BMI), preoperative peripheral oxygen saturation (SpO₂), haemoglobin, preoperative creatinine, American Society of Anesthesiologists (ASA) physical status, smoking and alcohol history, hypertension, preoperative pneumonia, emergency surgery, surgical type, surgical approach, surgical duration, fluid infusion volume, intraoperative blood loss, red blood cell (RBC) transfusion, vasoactive drug use, bronchial blocker use, left-sided OLV, nerve block, patient-controlled analgesia, lowest intraoperative SpO₂, and intraoperative hypoxaemia.

All patients were routinely monitored by electrocardiography, blood pressure measurement, and SpO₂ during mechanical ventilation under general anaesthesia. Mechanical ventilation was administered with 100% oxygen after intubation, as the operating room was not equipped with an air supply. Tidal volume was around 6–8 mL/kg, and PEEP was set around 0–10 cm H₂O in most OLV patients.

Preoperative anaemia was defined as haemoglobin below 130 g/L in men or below 120 g/L in women [8]. Surgical type was classified as intra-pulmonary (such as lobectomy) versus extra-pulmonary procedures (such as mediastinal surgery), and surgical approach as open thoracotomy versus video-assisted thoracoscopic surgery. RBC transfusion was analysed as any transfusion versus none. Intraoperative hypoxaemia was defined as a lowest recorded SpO₂ below 90%.

### Statistical analysis

Continuous variables were summarised as median [interquartile range] and categorical variables as n/available N (%). Continuous variables were assessed for normality using the Shapiro-Wilk test. As all continuous variables showed departures from normality, they were summarised as median [interquartile range] and compared using the Mann-Whitney U test. Categorical variables were compared using the Pearson chi-square test or Fisher exact test when expected cell counts were small. Standardised mean differences (SMDs) quantified the magnitude of distributional differences between patients with and without PPCs; because groups were defined by outcome rather than random allocation, SMDs were descriptive and were not interpreted as a balance diagnostic for randomisation [9, 10]. Descriptive, between-group, and univariable analyses used variable-specific available cases.

Univariable logistic regression described crude associations for all reported candidate variables. Variables with univariable *P* < 0.10 were considered for multivariable inclusion rather than entered automatically. Age, sex, and BMI were retained a priori on clinical grounds irrespective of their univariable P values. Intraoperative blood loss met the univariable threshold but was not carried forward because it represented a third correlated continuous marker of intraoperative exposure alongside surgical duration and fluid infusion volume; its Spearman correlations with these variables were 0.776 and 0.729, respectively. Its correlation with binary red blood cell transfusion was 0.355, so the exclusion was not attributed to blood loss-transfusion multicollinearity. Surgical duration and fluid infusion volume were retained because they represented duration of exposure and administered volume, while blood loss remained reported in the univariable analysis. The final model included 14 covariates: age, sex, BMI, ASA physical status at least III, preoperative anaemia, alcohol use, intra-pulmonary procedures, open thoracotomy, surgical duration, bronchial blocker use, RBC transfusion, fluid infusion volume, vasoactive drug use, and intraoperative hypoxaemia. Variance inflation factors (VIFs) were used to assess multicollinearity; VIFs below 5 were considered to indicate no important multicollinearity. With 211 PPC events, the primary model had 15.1 events per covariate [11].

Missingness was described for each variable. Little’s test was applied only to the nine variables with at least one missing value and assessed compatibility with missing completely at random (MCAR) [12]. Exploratory missingness-indicator analyses compared records with and without missing values on the outcome and selected observed covariates. The primary analysis used multiple imputation by chained equations (MICE) under a missing-at-random (MAR) working assumption [13, 14]. Predictive mean matching was used for continuous variables and logistic regression for binary variables. The outcome, all model covariates, and observed auxiliary variables included in the missing-data assessment were included as predictors; the outcome itself was not imputed. Fifty imputed datasets were generated with 30 iterations and random seed 20,250,705. Multivariable logistic regression coefficients and standard errors were combined using Rubin’s rules. These procedures cannot verify MAR or exclude missing not at random.

Two sensitivity analyses were fitted using the same covariate definitions: a complete-case 14-covariate model and a no-BMI MICE model containing the remaining 13 covariates. Adjusted ORs and 95% CIs were the primary association measures. Because PPCs were common, ORs were not interpreted as RRs [15]. Marginal standardisation of the Rubin-pooled model estimated complementary RRs and AMEs. For binary predictors, AMEs represented the mean absolute change in predicted PPC probability when the predictor changed from absent to present; for surgical duration, they represented a 1-hour increase. Ninety-five per cent CIs were obtained by 5000 parametric draws from the pooled coefficient covariance matrix using random seed 42.

Apparent discrimination was described by the area under the receiver operating characteristic curve (AUC), overall prediction error by the Brier score, and goodness of fit by the Hosmer-Lemeshow test. For MI analyses, these summaries were calculated from subject-level predicted probabilities averaged across the 50 imputed datasets. A non-significant Hosmer-Lemeshow result was not interpreted as proof of calibration, and none of these in-sample measures was treated as model validation. Two-sided *P* < 0.05 indicated statistical significance. Analyses were performed using R version 4.5.1; multiple imputation used the mice package version 3.19.0.

## Results

### Cohort characteristics and PPC occurrence

Of the 492 patients, 211 (42.9%) developed at least one PPC within the first 7 postoperative days (Supplementary Appendix S1).

The cohort was predominantly male (326/492, 66.3%) and Tibetan (470/492, 95.5%). Overall, 83 patients (16.9%) had ASA physical status at least III, 75/491 (15.3%) met the sex-specific definition of preoperative anaemia, 41 (8.3%) received a bronchial blocker, 28 (5.7%) received an RBC transfusion, and 90 (18.3%) underwent emergency surgery. Descriptive comparisons, exact denominators, P values, and SMDs are shown in Table 1.

**Table 1:** Baseline characteristics of patients undergoing thoracic surgery with one-lung ventilation at high altitude

Characteristic	Overall (*N* = 492)	No PPC (*n* = 281)	PPC (*n* = 211)	*P* value	SMD
Age, years	45.0 [28.8, 58.0]	46.0 [27.0, 58.0]	45.0 [31.5, 57.0]	0.557	0.056
Male sex	326/492 (66.3%)	181/281 (64.4%)	145/211 (68.7%)	0.317	0.091
BMI, kg/m²	21.3 [18.7, 24.6]	21.0 [18.6, 24.0]	21.4 [18.9, 25.0]	0.315	0.137
Tibetan ethnicity	470/492 (95.5%)	271/281 (96.4%)	199/211 (94.3%)	0.258	0.101
Hypertension	41/492 (8.3%)	25/281 (8.9%)	16/211 (7.6%)	0.602	0.048
Smoking history	134/492 (27.2%)	69/281 (24.6%)	65/211 (30.8%)	0.123	0.140
Alcohol use	141/492 (28.7%)	71/281 (25.3%)	70/211 (33.2%)	0.055	0.175
Preoperative pneumonia	40/492 (8.1%)	20/281 (7.1%)	20/211 (9.5%)	0.343	0.086
Preoperative SpO₂, %	90.0 [89.0, 93.0]	91.0 [89.0, 94.0]	90.0 [89.0, 92.0]	0.101	0.127
Haemoglobin, g/L	153.0 [135.0, 170.0]	150.5 [137.0, 166.0]	158.0 [130.0, 172.0]	0.243	0.024
Preoperative anaemia^a^	75/491 (15.3%)	31/280 (11.1%)	44/211 (20.9%)	0.003	0.269
Preoperative creatinine, µmol/L	65.0 [54.0, 75.0]	67.0 [57.0, 78.0]	61.0 [51.0, 72.0]	< 0.001	0.110
ASA physical status ≥ III ^b^	83/492 (16.9%)	27/281 (9.6%)	56/211 (26.5%)	< 0.001	0.451
Emergency surgery	90/492 (18.3%)	54/281 (19.2%)	36/211 (17.1%)	0.541	0.056
Surgical type^c^				**< 0.001**	**0.358**
Extra-pulmonary procedures	190/488 (38.9%)	88/279 (31.5%)	102/209 (48.8%)		
Intra-pulmonary procedures	298/488 (61.1%)	191/279 (68.5%)	107/209 (51.2%)		
Surgical approach^d^				**< 0.001**	**0.355**
VATS	388/488 (79.5%)	239/279 (85.7%)	149/209 (71.3%)		
Open thoracotomy	100/488 (20.5%)	40/279 (14.3%)	60/209 (28.7%)		
Surgical duration, min	125.0 [77.0, 210.0]	100.0 [65.0, 155.0]	170.0 [115.0, 265.0]	< 0.001	0.707
Bronchial blocker use	41/492 (8.3%)	15/281 (5.3%)	26/211 (12.3%)	0.006	0.248
Left-sided OLV	276/486 (56.8%)	153/278 (55.0%)	123/208 (59.1%)	0.367	0.083
Fluid infusion volume, mL	1300.0 [1000.0, 2000.0]	1000.0 [800.0, 1500.0]	1500.0 [1000.0, 2500.0]	< 0.001	0.619
Intraoperative blood loss, mL	100.0 [20.0, 200.0]	50.0 [20.0, 100.0]	150.0 [50.0, 300.0]	< 0.001	0.345
RBC transfusion^e^	28/492 (5.7%)	6/281 (2.1%)	22/211 (10.4%)	< 0.001	0.347
Vasoactive drug use	274/492 (55.7%)	144/281 (51.2%)	130/211 (61.6%)	0.022	0.210
Nerve block	79/492 (16.1%)	43/281 (15.3%)	36/211 (17.1%)	0.599	0.048
PCA	203/492 (41.3%)	118/281 (42.0%)	85/211 (40.3%)	0.703	0.035
Lowest intraoperative SpO₂, %	93.0 [87.0, 97.0]	95.0 [88.0, 98.0]	91.0 [85.0, 96.0]	< 0.001	0.397
Intraoperative hypoxaemia	169/492 (34.3%)	82/281 (29.2%)	87/211 (41.2%)	0.005	0.254

Data are median [interquartile range] for continuous variables and n/N (%) for categorical variables. Shapiro-Wilk testing indicated departures from normality for all continuous variables; continuous variables were therefore compared using the Mann-Whitney U test. Categorical variables were compared using Pearson’s chi-square test or Fisher’s exact test, as appropriate

^a^ Preoperative anaemia was defined as haemoglobin < 130 g/L in men or < 120 g/L in women

^b^ ASA physical status was assigned preoperatively by attending anaesthesiologists

^c^ Surgical type was classified as intra-pulmonary versus extra-pulmonary procedures; intra-pulmonary procedures included operations such as lobectomy, whereas extra-pulmonary procedures included operations such as mediastinal surgery

^d^ Surgical approach was classified as open thoracotomy versus video-assisted thoracoscopic surgery

^e^ RBC transfusion was defined as any recorded RBC transfusion volume > 0 mL

*Abbreviations*: *PPC* Postoperative pulmonary complication, *SMD* Standardised mean difference, *BMI* Body mass index, *SpO*₂ Peripheral oxygen saturation, *ASA* American Society of Anesthesiologists, *VATS* Video-assisted thoracoscopic surgery, *OLV* One-lung ventilation, *RBC* Red blood cell, *PCA* Patient-controlled analgesia

### Univariable logistic regression

Univariable results are presented in Table 2 for all reported candidate variables. Twelve variables had *P* < 0.10: ASA physical status at least III, preoperative anaemia, alcohol use, intra-pulmonary procedures, open thoracotomy, surgical duration, bronchial blocker use, RBC transfusion, fluid infusion volume, intraoperative blood loss, vasoactive drug use, and intraoperative hypoxaemia. Intraoperative blood loss was not carried forward because of the overlapping intraoperative-exposure information described above; the remaining 11 variables were retained. Age, sex, and BMI were additionally retained a priori, producing the 14-covariate model. Univariable associations were treated as crude descriptive evidence, not as causal effects.

**Table 2:** Univariable logistic regression analyses for postoperative pulmonary complications

Variable	OR (95% CI)	*P* value
Age, per 10-year increase	1.034 (0.929–1.151)	0.536
Male sex	1.214 (0.830–1.775)	0.318
BMI, kg/m²	1.030 (0.986–1.076)	0.180
Tibetan ethnicity	0.612 (0.259–1.445)	0.262
Hypertension	0.840 (0.437–1.617)	0.602
Smoking history	1.368 (0.918–2.039)	0.124
Alcohol use	**1.468 (0.991–2.175)**	**0.055**
Preoperative pneumonia	1.366 (0.715–2.610)	0.344
Preoperative SpO₂, %	0.967 (0.923–1.014)	0.165
Preoperative anaemia^a^	**2.116 (1.284–3.488)**	**0.003**
Preoperative creatinine, µmol/L	0.995 (0.986–1.004)	0.250
ASA physical status ≥ III	**3.399 (2.060–5.608)**	**< 0.001**
Emergency surgery	0.865 (0.543–1.377)	0.541
Intra-pulmonary procedures^b^	**0.483 (0.334–0.700)**	**< 0.001**
Open thoracotomy^c^	**2.406 (1.535–3.771)**	**< 0.001**
Surgical duration, per 1-hour increase	**1.471 (1.315–1.645)**	**< 0.001**
Bronchial blocker use	**2.492 (1.285–4.835)**	**0.007**
Left-sided OLV	1.182 (0.822–1.701)	0.367
Fluid infusion volume, per 100 mL	**1.071 (1.048–1.095)**	**< 0.001**
Intraoperative blood loss, per 100 mL increase	**1.140 (1.053–1.234)**	**0.001**
RBC transfusion^d^	**5.335 (2.123–13.407)**	**< 0.001**
Vasoactive drug use	**1.527 (1.062–2.195)**	**0.022**
Nerve block	1.139 (0.702–1.847)	0.599
Patient-controlled analgesia	0.932 (0.648–1.340)	0.703
Intraoperative hypoxaemia	**1.703 (1.169–2.480)**	**0.006**

PPC was the dependent variable. ORs for continuous variables correspond to the units shown in the table; BMI, preoperative SpO₂, and creatinine are reported per 1 kg/m², 1% point, and 1 µmol/L, respectively. Reference categories were female sex, non-Tibetan ethnicity, ASA physical status < III, no anaemia, no alcohol use, elective or time-limited surgery, extra-pulmonary procedures, VATS, and absence of the listed binary characteristic. Bold indicates univariable *P* < 0.10; these variables were considered, but not automatically entered, for multivariable modelling. Age, sex, and BMI were retained a priori. Intraoperative blood loss was not carried forward after redundancy review because of its correlations with surgical duration and fluid infusion volume (Spearman coefficients 0.776 and 0.729, respectively). Lowest intraoperative SpO₂ was reported descriptively but was not carried forward because it represents overlapping oxygenation information with the binary intraoperative hypoxaemia variable

^a^ Anaemia was defined as haemoglobin < 130 g/L in men or < 120 g/L in women

^b^ Intra-pulmonary procedures were compared with extra-pulmonary procedures

^c^ Open thoracotomy was compared with VATS

^d^ RBC transfusion denoted any recorded RBC transfusion volume > 0 mL

*Abbreviations*: *ASA* American Society of Anesthesiologists, *BMI* Body mass index, *CI* Confidence interval, *OLV* One-lung ventilation, *OR* Odds ratio, *PPC* Postoperative pulmonary complication, *RBC* Red blood cell, *SpO*₂ Peripheral oxygen saturation, *VATS* Video-assisted thoracoscopic surgery

### Primary multiple-imputation model

**Fig. 2:**
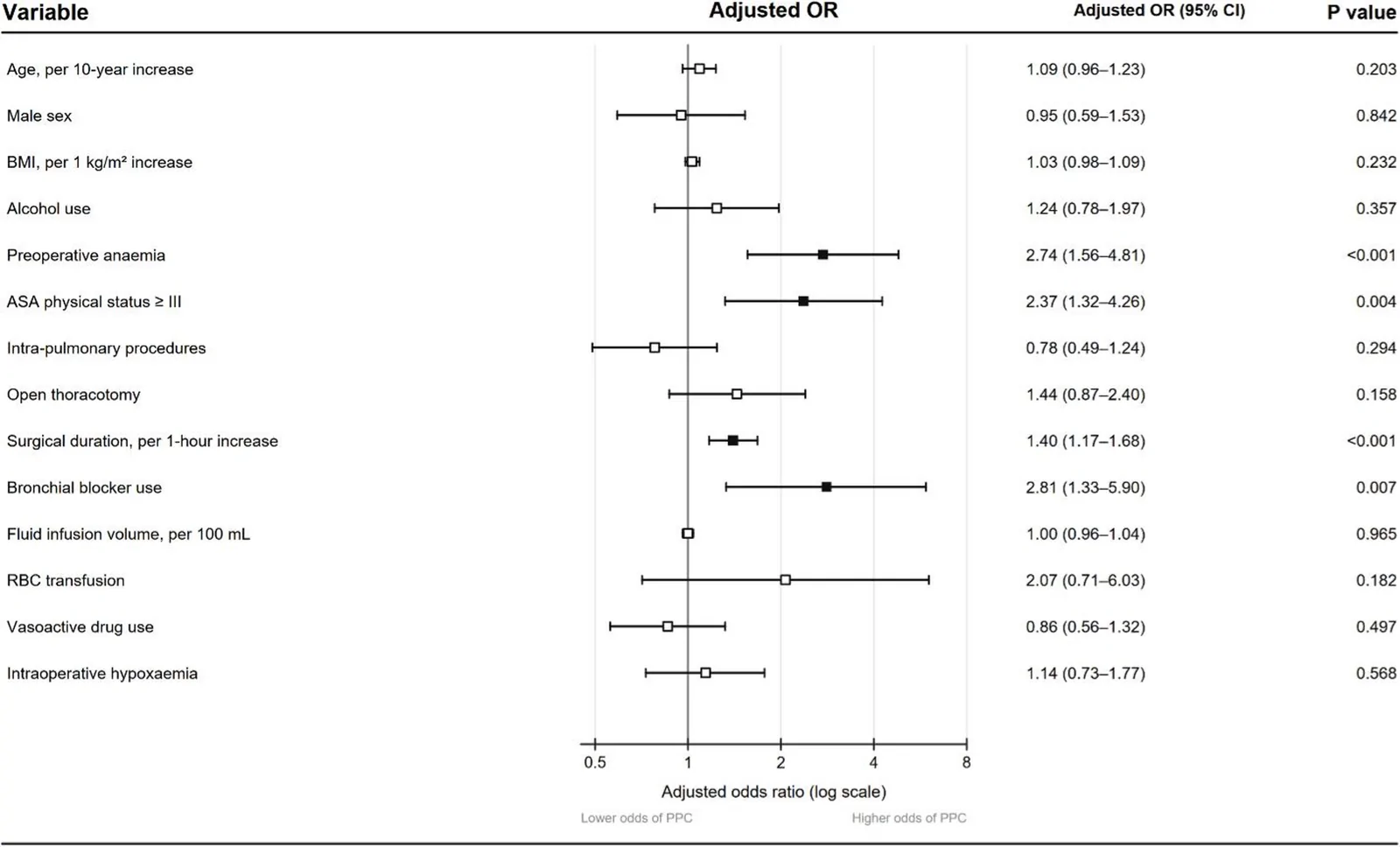
Forest plot of the primary multivariable logistic regression model. Filled squares indicate *P* < 0.05, open squares indicate *P* ≥ 0.05, horizontal lines indicate 95% CIs, and the vertical line indicates an adjusted OR of 1

The primary MICE model included all 492 patients and 211 PPC events. VIFs ranged from 1.05 to 2.76, indicating no important multicollinearity. After multivariable adjustment, ASA physical status at least III (OR 2.37, 95% CI 1.32–4.26; *P* = 0.004), preoperative anaemia (OR 2.74, 95% CI 1.56–4.81; *P* < 0.001), surgical duration per 1-hour increase (OR 1.40, 95% CI 1.17–1.68; *P* < 0.001), and bronchial blocker use (OR 2.81, 95% CI 1.33–5.90; *P* = 0.007) remained associated with PPCs. Other covariates did not meet the prespecified statistical-significance threshold after adjustment (Table 3; Fig. 2).

**Table 3:** Comparison of the primary and sensitivity multivariable logistic regression models

Variable	Primary MI OR (95% CI) (*n* = 492; 211 events)	*P* value	Complete-case OR (95% CI) (*n* = 398; 163 events)	*P* value	No-BMI MI OR (95% CI) (*n* = 492; 211 events)	*P* value
Age, per 10-year increase	1.09 (0.96–1.23)	0.203	1.10 (0.96–1.27)	0.180	1.06 (0.94–1.20)	0.333
Male sex	0.95 (0.59–1.53)	0.842	0.91 (0.54–1.56)	0.743	0.89 (0.56–1.40)	0.609
BMI, kg/m²	1.03 (0.98–1.09)	0.232	1.03 (0.98–1.09)	0.254	—	—
Alcohol use	1.24 (0.78–1.97)	0.357	1.51 (0.89–2.55)	0.129	1.27 (0.80–2.00)	0.314
Preoperative anaemia^a^	**2.74 (1.56–4.81)**	**< 0.001**	**2.26 (1.15–4.42)**	**0.018**	**2.55 (1.48–4.42)**	**< 0.001**
ASA physical status ≥ III	**2.37 (1.32–4.26)**	**0.004**	**2.88 (1.48–5.58)**	**0.002**	**2.45 (1.37–4.39)**	**0.003**
Intra-pulmonary procedures^b^	0.78 (0.49–1.24)	0.294	0.80 (0.47–1.34)	0.394	0.75 (0.48–1.17)	0.206
Open thoracotomy^c^	1.44 (0.87–2.40)	0.158	1.54 (0.87–2.72)	0.138	1.47 (0.89–2.44)	0.135
Surgical duration, per 1-hour increase	**1.40 (1.17–1.68)**	**< 0.001**	**1.37 (1.12–1.68)**	**0.002**	**1.40 (1.17–1.68)**	**< 0.001**
Bronchial blocker use	**2.81 (1.33–5.90)**	**0.007**	**2.89 (1.29–6.48)**	**0.010**	**2.79 (1.33–5.86)**	**0.007**
Fluid infusion volume, per 100 mL	1.00 (0.96–1.04)	0.965	1.00 (0.96–1.04)	0.959	1.00 (0.96–1.03)	0.898
RBC transfusion^d^	2.07 (0.71–6.03)	0.182	2.39 (0.65–8.77)	0.188	1.99 (0.68–5.78)	0.207
Vasoactive drug use	0.86 (0.56–1.32)	0.497	0.94 (0.58–1.52)	0.787	0.88 (0.57–1.35)	0.555
Intraoperative hypoxaemia	1.14 (0.73–1.77)	0.568	1.16 (0.70–1.92)	0.577	1.15 (0.74–1.79)	0.543

Values are adjusted ORs (95% CIs). The primary MI model included 14 covariates; the complete-case model included the same 14 covariates; and the no-BMI MI model excluded BMI and included the remaining 13 covariates. BMI was modelled per 1 kg/m². Missing values were handled using multiple imputation by chained equations with 50 imputed datasets, 30 iterations, and random seed 20250705; continuous variables were imputed using predictive mean matching and binary variables using logistic regression. Estimates were combined using Rubin’s rules. VIFs ranged from 1.05 to 2.76. A dash indicates that BMI was omitted. Bold indicates *P* < 0.05

^a^ Anaemia was defined as haemoglobin < 130 g/L in men or < 120 g/L in women

^b^ Intra-pulmonary procedures were compared with extra-pulmonary procedures

^c^ Open thoracotomy was compared with VATS

^d^ RBC transfusion denoted any recorded RBC transfusion volume > 0 mL

*Abbreviations*: *BMI* Body mass index, *CI* Confidence interval, *MI* Multiple imputation, *OR* Odds ratio, *RBC* Red blood cell, *VATS* Video-assisted thoracoscopic surgery, *VIF* Variance inflation factor

The apparent AUC was 0.765, the Brier score was 0.1962, and the Hosmer-Lemeshow P value was 0.295 (Table 4). These measures describe in-sample model performance and do not constitute internal or external validation.

**Table 4:** Apparent in-sample performance of the primary and sensitivity models

Performance measure	Primary MI (*n* = 492; 211 events)	Complete-case (*n* = 398; 163 events)	No-BMI MI (*n* = 492; 211 events)
AUC (c-statistic)	0.765	0.774	0.764
Brier score	0.1962	0.1905	0.1970
Hosmer-Lemeshow test P value	0.295	0.251	0.351

Performance estimates are apparent in-sample measures. For MI models, subject-level predicted probabilities were averaged across imputations

*Abbreviations*: *AUC* Area under the receiver operating characteristic curve, *MI* Multiple imputation

### Missing data and sensitivity analyses

BMI had the highest missingness (89/492, 18.1%) (Supplementary Table S1). Little’s MCAR test was applied only to the nine variables with at least one missing value (BMI, intraoperative blood loss, lowest intraoperative SpO₂, left-sided OLV, preoperative SpO₂, surgical type, surgical approach, preoperative anaemia, and fluid infusion volume) and yielded χ²=116.81, df = 96, and *P* = 0.0732. This was compatible with MCAR but did not prove MCAR; MAR and MNAR could not be excluded. In exploratory missingness-indicator analyses, BMI missingness showed weak associations with PPC status (Pearson’s chi-square test, *P* = 0.064) and surgical duration (Wilcoxon rank-sum test, *P* = 0.058), while missingness of surgical type and approach was associated with red blood cell transfusion (Fisher’s exact test, *P* = 0.017). These observations supported conditioning the imputation model on observed variables, but they did not prove MAR; a missing-not-at-random mechanism could not be excluded.

The complete-case model included 398 patients and 163 PPC events. The no-BMI MICE model included 492 patients and 211 events. The directions and statistical significance of ASA physical status at least III, preoperative anaemia, surgical duration, and bronchial blocker use were consistent across the three analyses (Table 3). Apparent AUCs were 0.765, 0.774, and 0.764; Brier scores were 0.1962, 0.1905, and 0.1970; and Hosmer-Lemeshow P values were 0.295, 0.251, and 0.351 for the primary MICE, complete-case, and no-BMI MICE models, respectively (Table 4).

Marginal standardisation of the primary model yielded RRs of 1.45 (95% CI 1.13–1.79) for ASA physical status at least III, 1.52 (95% CI 1.22–1.82) for preoperative anaemia, 1.16 (95% CI 1.08–1.24) per 1-hour increase in surgical duration, and 1.51 (95% CI 1.13–1.87) for bronchial blocker use. Corresponding AMEs were + 18.1 (95% CI + 5.3 to + 29.8), + 20.6 (95% CI + 9.0 to + 31.1), + 6.8 (95% CI + 3.2 to + 10.0), and + 21.0 (95% CI + 5.6 to + 34.3) percentage points. Marginal-effect estimates for these principal associations are reported in Supplementary Table S2.

## Discussion

In this single-centre high-altitude thoracic OLV cohort, 42.9% of patients developed at least one PPC within the first 7 postoperative days, predominantly atelectasis or pneumonia. In the primary MICE analysis, ASA physical status at least III, preoperative anaemia, longer surgical duration, and bronchial blocker use remained associated with PPCs after adjustment, with consistent directions in the complete-case and no-BMI MICE analyses. These observational estimates neither isolate an effect of altitude nor establish that modifying haemoglobin status, operative duration, or lung-isolation strategy would reduce PPCs.

The observed PPC incidence was higher than in some broader surgical cohorts [1–3], but direct comparisons are limited by differences in endpoint definitions, surgical populations, ascertainment, and follow-up. Respiratory failure and reintubation were rare in this cohort, indicating that the composite incidence reflected a burden dominated by atelectasis and pneumonia.

Lower inspired oxygen partial pressure is an important physiological feature of the high-altitude setting [6], but this study had no low-altitude comparator and cannot quantify an independent altitude effect. During OLV, gas exchange depends predominantly on the non-operative lung, while general anaesthesia, neuromuscular blockade, lateral positioning, lung collapse, and surgical manipulation may reduce functional residual capacity, promote atelectasis, and alter ventilation-perfusion matching [4, 5]. Baseline physiological reserve, oxygen-carrying capacity, exposure duration, and lung-isolation complexity may therefore jointly influence PPC occurrence; these are mechanistic interpretations rather than tested causal pathways.

Although ambient hypobaric hypoxia at high altitude may be associated with lower baseline SpO₂, during one-lung ventilation patients routinely receive an inspired oxygen fraction of 1.0 via mechanical ventilation, so intraoperative SpO₂ is maintained substantially above ambient baseline. We therefore retained SpO₂ <90% as the threshold for intraoperative hypoxaemia because it is widely used in the perioperative and one-lung ventilation literature [4, 5], corresponds to a clinically important level of oxygen desaturation even under 100% oxygen delivery, and maintains comparability with previous studies.

ASA physical status at least III remained associated with PPCs across all analyses. Its adjusted OR was 2.37, marginally standardised RR 1.45, and AME + 18.1% points. ASA physical status summarises systemic disease burden and physiological reserve rather than a single mechanism. In thoracic OLV patients, a higher ASA category may mark cardiopulmonary disease, infection or inflammation, nutritional or metabolic vulnerability, and reduced tolerance of perioperative atelectasis, secretion retention, and oxygenation instability. The finding may help identify patients who warrant closer perioperative assessment, but it does not identify a specific intervention that would reduce PPCs.

Preoperative anaemia was associated with an adjusted OR of 2.74, RR of 1.52, and AME of + 20.6% points. Reduced haemoglobin may lower arterial oxygen content even when SpO₂ is unchanged, thereby narrowing oxygen-delivery reserve during OLV. Anaemia may also mark nutritional deficiency, chronic inflammation, renal disease, or other systemic vulnerability. WHO recommends considering residential altitude when haemoglobin is used to diagnose anaemia, but notes that adjustments above 3000 m are based primarily on data from Central and South America and that region- or ethnicity-specific adjustments may be warranted at very high elevations [8]. This limitation is relevant to Lhasa because the haemoglobin response to altitude varies among populations, and Tibetan and Andean highlanders show distinct adaptive patterns; compared with Andean highlanders, Tibetans generally show a smaller haemoglobin increase together with stronger ventilatory adaptation [16, 17]. Individual residence altitude and duration of high-altitude exposure were unavailable in this retrospective dataset, so applying a single correction based on the hospital altitude would have imposed an uncertain population-level adjustment on all patients. We therefore used the conventional sex-specific thresholds as a reproducible clinical classification rather than as validated altitude-adjusted physiological cutoffs. The finding identifies a clinically relevant association but cannot determine whether correcting anaemia would reduce PPCs.

For each 1-hour increase in surgical duration, the adjusted OR was 1.40, RR 1.16, and AME + 6.8% points. Surgical duration may integrate procedural complexity, tissue injury, OLV and mechanical-ventilation exposure, anaesthetic exposure, and fluid accumulation. Because exact OLV duration and granular ventilation variables were unavailable, their relative contributions could not be separated. Surgical duration should therefore be viewed as a proxy for cumulative perioperative exposure rather than an isolated causal factor.

Bronchial blocker use was associated with an adjusted OR of 2.81, RR of 1.51, and AME of + 21.0% points. Only 41 patients received a bronchial blocker, and device selection was not random. Bronchial blockers may be selected for difficult airways, particular airway anatomy, or specific surgical requirements; suctioning and operative-lung deflation may also differ from double-lumen tubes. Previous comparative studies have reported inconsistent pulmonary outcomes [18–21]. Because device-selection rationale, bronchoscopic repositioning, lung-collapse quality, and contamination-control requirements were unavailable, the data cannot distinguish a device effect from confounding by indication. The estimate should therefore be interpreted as an association observed in a small, selectively exposed subgroup rather than evidence that bronchial blockers cause PPCs.

This study has three main limitations. First, it was a single-centre retrospective cohort without a low-altitude comparison group; it cannot establish an independent causal effect of high altitude or separate altitude, institutional practice, and case mix. Second, bronchial blocker exposure and red blood cell transfusion were sparse and non-random, and the surgical categories were clinically heterogeneous; confounding by indication and residual confounding remain likely. Third, pulmonary function, arterial blood gases, exact PEEP and tidal volume, driving pressures, recruitment manoeuvres, OLV duration, lung-collapse quality, and bronchoscopic adjustments were unavailable. Missing-data analyses relied on an untestable MAR working assumption, and endpoints were retrospectively ascertained without independent blinded adjudication.

## Conclusions

In this single-centre high-altitude thoracic OLV cohort, PPCs occurred in 42.9% of patients and were predominantly atelectasis and pneumonia. After multivariable adjustment, ASA physical status ≥ III, preoperative anaemia, longer surgical duration, and bronchial blocker use remained associated with PPCs. The bronchial-blocker estimate is particularly susceptible to confounding by indication. In the absence of a low-altitude comparator and granular ventilation data, these findings should be regarded as observational signals requiring confirmation in multicentre prospective studies.

## Supplementary Information

Supplementary Material 1.

## Data Availability

The datasets analyzed during the current study are not publicly available because they contain clinical information subject to institutional privacy restrictions, but may be available from the corresponding author on reasonable request and with appropriate institutional approval.

## Abbreviations

AME: Average marginal effect
ARDS: Acute respiratory distress syndrome
ASA: American Society of Anesthesiologists
AUC: Area under the receiver operating characteristic curve
BMI: Body mass index
CI: Confidence interval
FiO₂: Fraction of inspired oxygen
MAR: Missing at random
MCAR: Missing completely at random
MICE: Multiple imputation by chained equations
OLV: One-lung ventilation
OR: Odds ratio
PEEP: Positive end-expiratory pressure
PPCs: Postoperative pulmonary complications
RR: Risk ratio
SMD: Standardised mean difference
SpO₂: Peripheral oxygen saturation
STROBE: Strengthening the Reporting of Observational Studies in Epidemiology
VIF: Variance inflation factor
