# Supplementary material for: Postoperative pulmonary complications after high-altitude thoracic surgery requiring one-lung ventilation: a single-centre retrospective cohort study
Source: BMC Anesthesiol. 2026 Aug 8;26:627. doi: 10.1186/s12871-026-04139-6 (PMC13682143; doi:10.1186/s12871-026-04139-6)
Supplement: Supplementary file 1 [file 12871_2026_4139_MOESM1_ESM.docx]

# Supplementary Material

Postoperative Pulmonary Complications After High-Altitude Thoracic Surgery Requiring One-Lung Ventilation: A Single-Centre Retrospective Cohort Study

## Appendix S1. Operational definitions of postoperative pulmonary complication components

The primary outcome was an in-hospital composite of postoperative pulmonary complications (PPCs). A patient was classified as having a PPC if at least one of the six components listed below occurred within the first 7 postoperative days. The definitions of the PPC components were based principally on Douville et al. [1]. The radiographic definition of atelectasis was informed by Canet et al. [2], with the study-specific modification described in the table footnote.

Pleural effusion and pneumothorax were not included in the primary composite. Pre-existing pneumonia was treated only as a baseline covariate, whereas newly identified postoperative pneumonia was treated as an outcome component. PPC components were retrospectively ascertained from the available clinical records and were not independently adjudicated.

| **Component** | **Operational definition** | **Observed cases, n (%)** | **Reference** |
| --- | --- | --- | --- |
| Respiratory failure | Postoperative respiratory failure requiring mechanical ventilation and/or reintubation. | 1 (0.2%) | [1] |
| Pneumonia | Postoperative pneumonia meeting at least three of five criteria: fever, leukocytosis, a new infiltrate on chest imaging, a positive sputum culture, or treatment with antibiotics. | 113 (23.0%) | [1] |
| Acute respiratory distress syndrome (ARDS) | Acute-onset respiratory distress meeting all of the following criteria: PaO₂/FiO₂ <200, irrespective of PEEP level; bilateral infiltrates on chest radiography; pulmonary artery occlusion pressure <18 mmHg or no clinical evidence of left atrial hypertension; and the presence of a compatible risk factor. All criteria were required. | 0 (0.0%) | [1] |
| Ventilatory support for >48 h | Initial postoperative ventilatory support lasting more than 48 h. Support ended at tracheal extubation or, for patients with a tracheostomy, when ventilator dependence ceased. | 25 (5.1%) | [1] |
| Reintubation | Repeat tracheal intubation after postoperative extubation. | 1 (0.2%) | [1] |
| Atelectasis* | Postoperative atelectasis detected on chest radiography or computed tomography, defined as lung opacification with a shift of the mediastinum, hilum, or hemidiaphragm toward the affected area and compensatory overinflation of the adjacent non-atelectatic lung. | 141 (28.7%) | [2] |

*The PPC definitions were based principally on Douville et al. [1]. Unlike Douville et al., the present study classified atelectasis identified on chest imaging as a PPC without requiring bronchoscopic confirmation because bronchoscopy data could not be reliably ascertained from the electronic medical records. The radiographic criteria for atelectasis were informed by Canet et al. [2].

## Supplementary Table S1. Missing data in variables used for analysis

| **Variable** | **Missing, n** | **Missing, %** |
| --- | --- | --- |
| Body mass index | 89 | 18.1 |
| Intraoperative blood loss | 20 | 4.1 |
| Lowest intraoperative SpO₂ | 18 | 3.7 |
| Left-sided one-lung ventilation | 6 | 1.2 |
| Preoperative SpO₂ | 5 | 1.0 |
| Surgical type | 4 | 0.8 |
| Surgical approach | 4 | 0.8 |
| Preoperative anaemia | 1 | 0.2 |
| Fluid infusion volume | 1 | 0.2 |

Little's test was applied only to the nine variables with at least one missing value (BMI, intraoperative blood loss, lowest intraoperative SpO₂, left-sided OLV, preoperative SpO₂, surgical type, surgical approach, preoperative anaemia, and fluid infusion volume) and yielded χ²=116.81, df=96, and P=0.0732. This was compatible with MCAR but did not prove MCAR; MAR and MNAR could not be excluded. Multiple imputation by chained equations was therefore implemented under a missing-at-random working assumption conditional on observed variables; a missing-not-at-random mechanism could not be excluded. The outcome and all model covariates were included in the imputation model, but the outcome itself was not imputed. Fifty imputed datasets were generated using 30 iterations and random seed 20250705. Predictive mean matching was used for continuous variables and logistic regression for binary variables. Multivariable estimates and standard errors were combined using Rubin's rules.

## Supplementary Table S2. Marginally standardised risk ratios and average marginal effects for the principal associations in the primary model

| **Variable** | **Adjusted OR (95% CI)** | **RR (95% CI)** | **AME, percentage points (95% CI)** |
| --- | --- | --- | --- |
| ASA physical status ≥ III | 2.37 (1.32-4.26) | 1.45 (1.13-1.79) | +18.1 (+5.3 to +29.8) |
| Preoperative anaemia | 2.74 (1.56-4.81) | 1.52 (1.22-1.82) | +20.6 (+9.0 to +31.1) |
| Surgical duration, per 1-hour increase | 1.40 (1.17-1.68) | 1.16 (1.08-1.24) | +6.8 (+3.2 to +10.0) |
| Bronchial blocker use | 2.81 (1.33-5.90) | 1.51 (1.13-1.87) | +21.0 (+5.6 to +34.3) |

Risk ratios (RRs) and average marginal effects (AMEs) were obtained by marginal standardisation of the Rubin-pooled primary model. Confidence intervals were estimated from 5000 parametric draws from the pooled coefficient covariance matrix using random seed 42. For binary predictors, the AME is the mean absolute change in predicted PPC probability when the predictor changes from absent to present. For surgical duration, the contrast is a 1-hour increase. AMEs are reported in percentage points. These measures complement rather than replace the adjusted odds ratios.

## Supplementary Table S3. Multiple-imputation specification for variables with missing values

| **Variable** | **Variable type** | **Imputation method** |
| --- | --- | --- |
| Body mass index | Continuous | Predictive mean matching |
| Preoperative anaemia | Binary | Logistic regression |
| Surgical type | Binary | Logistic regression |
| Surgical approach | Binary | Logistic regression |
| Fluid infusion volume | Continuous | Predictive mean matching |
| Left-sided one-lung ventilation | Binary | Logistic regression |
| Preoperative SpO₂ | Continuous | Predictive mean matching |
| Intraoperative blood loss | Continuous | Predictive mean matching |
| Lowest intraoperative SpO₂ | Continuous | Predictive mean matching |

Abbreviations: AME, average marginal effect; CI, confidence interval; MICE, multiple imputation by chained equations; OR, odds ratio; PPC, postoperative pulmonary complication; RR, risk ratio.

# References

1. Douville NJ, Smolkin ME, Naik BI, et al. Association between inspired oxygen fraction and development of postoperative pulmonary complications in thoracic surgery: a multicentre retrospective cohort study. Br J Anaesth. 2024;133(5):1073-1084. doi:10.1016/j.bja.2024.08.005.

2. Canet J, Gallart L, Gomar C, et al. Prediction of postoperative pulmonary complications in a population-based surgical cohort. Anesthesiology. 2010;113(6):1338-1350. doi:10.1097/ALN.0b013e3181fc6e0a.
